# On the Geodesic Distance in Shapes *K*-means Clustering

**DOI:** 10.3390/e20090647

**Published:** 2018-08-29

**Authors:** Stefano Antonio Gattone, Angela De Sanctis, Stéphane Puechmorel, Florence Nicol

**Affiliations:** 1 Department of Philosophical, Pedagogical and Economic-Quantitative Sciences, University “G. d’Annunzio” of Chieti-Pescara, 66100 Chieti, Italy; 2 Department of Business Economics, University “G. D’Annunzio” of Chieti-Pescara, 65127 Pescara, Italy; 3 Ecole Nationale de l’aviation Civile (ENAC), Université Fédérale de Toulouse, FR-31055 Toulouse CEDEX, France

**Keywords:** Shape Analysis, clustering, *K*-means algorithm, Fisher-Rao metric, wasserstein distance

## Abstract

In this paper, the problem of clustering rotationally invariant shapes is studied and a solution using Information Geometry tools is provided. Landmarks of a complex shape are defined as probability densities in a statistical manifold. Then, in the setting of shapes clustering through a *K*-means algorithm, the discriminative power of two different shapes distances are evaluated. The first, derived from Fisher–Rao metric, is related with the minimization of information in the Fisher sense and the other is derived from the Wasserstein distance which measures the minimal transportation cost. A modification of the *K*-means algorithm is also proposed which allows the variances to vary not only among the landmarks but also among the clusters.

## 1. Introduction

Shapes clustering is of interest in various fields such as geometric morphometrics, computer vision and medical imaging. In the clustering of shapes, it is important to select an appropriate measurement of distance among observations. In particular, we are interested in classifying shapes which derive from complex systems as expression of self-organization phenomenon. We consider objects whose shapes are based on landmarks [1,2,3]. These objects can be obtained by medical imaging procedures, curves defined by manually or automatically assigned feature points or by a discrete sampling of the object contours.

Since the shape space is invariant under similarity transformations, that is translations, rotations and scaling, the Euclidean distance on such a space is not really meaningful. In Shape Analysis [4], to apply standard clustering algorithms to planar shapes, the Euclidean metric has to be replaced by the metric of the shape space. Examples were provided in References [5,6], where the Procrustes distance was integrated in standard clustering algorithms such as the *K*-means. Similarly, Lele and Richtsmeier [7] applied standard hierarchical or *K*-means clustering using dissimilarity measures based on the inter-landmark distances. In a model-based clustering framework, Huang and Zhu [8] and Kume and Welling [9] developed a mixture model of offset-normal shape distributions.

In Shape Analysis, it is common to assume that the landmark coordinates have an isotropic covariance structure [4]. To relax the isotropic assumption, a bivariate Gaussian model was proposed to describe the landmarks of a planar shape [10,11], where the means are the landmark geometric coordinates and capture uncertainties that arise in the landmark placement while the variances derive from the natural variability across the population of shapes. The novelty of this shape representation is given by the fact that variances are considered as additional coordinates for the landmarks of a shape. According to Information Geometry, the space of bivariate Gaussian densities is considered as a statistical manifold [12,13] with the local coordinates given by the model parameters. In this way, distances between landmarks can be defined using the geodesic distances induced by different Riemannian metrics.

In this paper, we consider the Fisher–Rao and the Wasserstein metrics as Riemannian metrics on the statistical manifold of the Gaussian densities. The geodesic distance induced by the Fisher–Rao metric is related to the minimization of information in the Fisher sense while the Wasserstein distance is related to the minimal transportation cost. Applications of geodesics to shape clustering techniques have also been provided in a landmark-free context [14,15].

As is well known, any hierarchical clustering algorithm uses as input the pairwise distances of all possible pairs of objects under study. Using the geodesic distances induced by Wasserstein and Fisher–Rao metrics, in References [10,11], a hierarchical clustering algorithm which allows the variances to vary among the landmarks was proposed.

In this paper, the discriminative power of these shapes distances is evaluated in the setting of shapes *K*-means clustering which is easier to implement and computationally faster. Furthermore, a modification of the *K*-means algorithm is proposed which allows the variances to vary not only among the landmarks but also among the clusters. The simulation results show that the proposed algorithm is able to cope with the effects of anisotropy in the landmark variances across different clusters.

## 2. Geometrical Structures for a Manifold of Probability Distributions

We call “manifold” a geometric object which is locally Euclidean then described by local coordinates. Manifolds can be used to study patterns from complex systems. Since pattern recognition essentially relies on quantitative assessment of the proximity of points, for the comparison of patterns, we need a well-suited similarity measure (distance or divergence). From Differential Geometry, we know that a Riemannian metric on a differential manifold *X* is induced by a metric matrix *g*, which defines an inner product on every tangent space of the manifold as follows: $\langle u,v\rangle =u^{T}g_{ij}v$ with associated norm $∥u∥=\sqrt{\langle u,u\rangle }$. Then, the distance between two points P,Q of the manifold is given by the minimum of the lengths of all the piecewise smooth paths γ joining these two points. Precisely, the length of a path is calculated using the inner product,

$$\mathrm{Length}\;\mathrm{of}\;\gamma =\int ∥\gamma^{\prime }\left(t\right)∥dt$$

thus

$$d\left(P,Q\right)=min_{\gamma }\left\{\mathrm{Length}\;\mathrm{of}\;\gamma \right\}.$$

A curve that encompasses this shortest path is called a Riemannian geodesic and the previous distance is named geodesic distance. We remark that in general the concept of geodesic is related to connections defined on a manifold. If a connection is not Riemannian, then a geodesic is different from a shortest path.

Probability theory, in the presence of non-deterministic phenomena, provides a natural description of the raw data. Each measurement *x* is regarded as a sample from an underlying probability distribution of the measurement characterized by its probability density function p(x/θ). Measurements described by the distribution parameters, θ, may contain more information than a measurement expressed as a value and an associated error bar. Therefore, we apply pattern recognition methods directly in the space of probability distributions. Let P be a family of probability density functions p(x∣θ) parameterized by $\theta \in R^{k}$. It is well known that we can endow it with a structure of manifold, called statistical manifold, whose local coordinates are the parameters of the family. As an example, we consider the family of *p*-variate Gaussian densities:

$$f\left(x|\theta =\left(\mu ,\Sigma \right)\right)={\left(2\pi \right)}^{-\frac{p}{2}}{\left(\det\Sigma \right)}^{-\frac{1}{2}}\exp\left\{-\frac{1}{2}{\left(x-\mu \right)}^{T}\Sigma^{-1}\left(x-\mu \right)\right\}$$

where $x={\left(x_{1},x_{2},\cdots ,x_{p}\right)}^{T}$, $\mu ={\left(\mu_{1},\mu_{2},\cdots \mu_{p}\right)}^{T}$ is the mean vector and Σ the covariance matrix. Note that the parameter space has dimension $k=p+\frac{p(p+1)}{2}$. In particular, we are interested in the case p=2.

Two geometrical structures have been extensively studied for a manifold of probability distributions. One is based on the Fisher information metric (Fisher–Rao metric), which is invariant under reversible transformations of random variables, while the other is based on the Wasserstein distance of optimal transportation, which reflects the structure of the distance between random variables.

In the statistical manifold of bivariate Gaussian densities, we consider these two different Riemannian metrics which in turn induce two types of geodesic distances.

### Fisher–Rao Metric for Gaussian Densities

The geometry of the Gaussian manifold endowed with the Fisher–Rao metric was intensively studied in References [16,17]. To avoid considering manifolds with boundaries, it is convenient to assume that all densities are non-degenerate, thus the covariance matrices are invertible. In this case, one can define the manifold of *n*-dimensional Gaussian densities as the set $R^{n}\times R^{n(n+1)/2}=R^{n+n(n+1)/2}$ with local charts given by the obvious identification $N_{n}\left(\mu ,\Sigma \right)\mapsto \left(\mu_{i,i=1\cdots n},\sigma_{ij,i=1\cdots n,j\leq n}\right)$, where the $\sigma_{ij}$ are the elements of the matrix Σ. A tangent vector at a point (μ,Σ) of the manifold is just a vector from $R^{n+n(n+1)/2}$. While quite tractable, this choice of parameterization does not give any insight about the structure of the manifold. A more enlightening approach is obtained by considering groups of transformations, as detailed below.

Let ${\mathrm{symm}}^{+}\left(n\right)$ be a group of symmetric positive definite matrices of size n×n endowed with the product [18]:

(1) $$\left(A,B\right)\mapsto A\circ B=A^{1/2}BA^{1/2}$$

and let us denote, using a common abuse of notation, the group of translations of $R^{n}$ also by $R^{n}$.

Now, define the group G(n) as the semi-direct product:

(2) $$G\left(n\right)={\mathrm{symm}}^{+}\left(n\right){⋉}_{\rho }R^{n}$$

where the action ρ of ${\mathrm{symm}}^{+}\left(n\right)$ on $R^{n}$ is given by left multiplication with the square root of the matrix, namely:

(3) $$\rho \left(A\right)u=A^{1/2}u,A\in {\mathrm{symm}}^{+}\left(n\right),u\in R^{n}$$

In the sequel, we are dropping the ρ subscript in the semi-direct product and assume it implicitly.

An element in G(n) can be represented as a couple (A,u) with $A\in {\mathrm{symm}}^{+}\left(n\right),u\in R^{n}$. The group product is obtained from the action ρ as $\left(A,u\right)\cdot \left(B,v\right)=\left(A^{1/2}BA^{1/2},A^{1/2}v+u\right)$.

The inverse of an element (A,u) is given by $\left(A^{-1},-A^{-1/2}u\right)$. The group G(n) is a Lie group with Lie algebra $g\left(n\right)={\mathrm{symm}}^{+}\left(n\right)⊕R^{n}$ with ${\mathrm{symm}}^{+}\left(n\right)$ the vector space of symmetric matrices. Finally, the left translation by an element (A,u) is the mapping:

(4) $$\left(B,v\right)\mapsto L_{\left(A,u\right)}\left(B,v\right)=\left(A,u\right)\cdot \left(B,v\right)$$

Being an affine map, its derivative is its linear part. The Frobenius inner product on the space of square matrices of dimension *n*, defined as $\langle A,B\rangle =\mathrm{tr}\left(A^{t}B\right)=\mathrm{tr}\left(AB^{t}\right)$, jointly with the standard euclidean inner product on $R^{n}$, induces a left invariant metric by:

(5) $$\langle \langle {\;\;\left(X,\eta \right),\left(Y,\xi \right)\rangle \rangle \;\;}_{\left(A,u\right)}=K\mathrm{tr}\left(A^{-1/2}XA^{-1}YA^{-1/2}\right)+\eta_{1}^{t}A^{-1}\eta_{1}$$

where (X,η),(Y,ξ) are tangent vectors to G(n) at (A,u) and K>0 is a fixed scaling factor that may be arbitrary chosen to balance the relative contributions of the matrix part and the translation part.

It turns out that the metric obtained that way is exactly the Fisher–Rao metric on the manifold of multivariate Gaussian densities. Using the notations of Skovgaard [16], the length element of the Fisher–Rao metric $g_{F}$ is:

(6) $$ds^{2}=\frac{1}{2}\mathrm{tr}\left(\Sigma^{-1}X\Sigma^{-1}X\right)+\eta^{t}\Sigma^{-1}\eta$$

with (X,η) a tangent vector at (Σ,μ).

The expression of $ds^{2}$ is the one of a warped product metric [19], which allows some simplifications when computing the geodesics between two densities with same means.

A closed form for the geodesic distance between two densities with diagonal covariance matrices may also be obtained as follows [17]:

(7) $$\begin{array}{cc} d_{F}\left(\theta ,\theta^{\prime }\right) & =\sqrt{2\sum_{i=1}^{2}{\left(\ln\frac{|\left(\frac{\mu_{i}}{\sqrt{2}},\sigma_{i}\right)-\left(\frac{\mu_{i}^{\prime }}{\sqrt{2}},-\sigma_{i}^{\prime }\right)\left|+\right|\left(\frac{\mu_{i}}{\sqrt{2}},\sigma_{i}\right)-\left(\frac{\mu_{i}^{\prime }}{\sqrt{2}},\sigma_{i}^{\prime }\right)|}{|\left(\frac{\mu_{i}}{\sqrt{2}},\sigma_{i}\right)-\left(\frac{\mu_{i}^{\prime }}{\sqrt{2}},-\sigma_{i}^{\prime }\right)\left|-\right|\left(\frac{\mu_{i}}{\sqrt{2}},\sigma_{i}\right)-\left(\frac{\mu_{i}^{\prime }}{\sqrt{2}},\sigma_{i}^{\prime }\right)|}\right)}^{2}} \end{array}$$

where θ=(μ,Σ) with $\mu =(\mu_{1},\mu_{2})$ and $\Sigma =\mathrm{diag}(\sigma_{1}^{2},\sigma_{2}^{2})$, $\theta^{\prime }=\left(\mu^{\prime },\Sigma^{\prime }\right)$ with $\mu^{\prime }=\left(\mu_{1}^{\prime },\mu_{2}^{\prime }\right)$ and $\Sigma^{\prime }=\mathrm{diag}\left({\left(\sigma_{1}^{\prime }\right)}^{2},{\left(\sigma_{2}^{\prime }\right)}^{2}\right)$.

For general Gaussian densities with Σ any symmetric positive definite covariance matrix, a closed form for the geodesic distance is not known and one has to solve numerically a system of differential equations:

(8) $$\begin{array}{c} D_{tt}\mu -D_{t}\Sigma \Sigma^{-1}D_{t}\mu =0 \end{array}$$

(9) $$\begin{array}{c} D_{tt}\Sigma +D_{t}\mu D_{t}\mu^{t}-D_{t}\Sigma \Sigma^{-1}D_{t}\Sigma =0 \end{array}$$

where the expression $D_{t}$ (respectively, $D_{tt}$) stands for derivative (respectively, second derivative) with respect to *t*. A geodesic between two densities can be found by a shooting approach, which starts with one density as an initial condition to the system in Equation (8) and iteratively adjusts the initial speed vector of the curve so as to reduce the distance to the target density until the desired accuracy is reached. A collocation algorithm can also be used, and is a common choice for solving ordinary differential equations with boundary conditions. It is generally more stable than the shooting method, but may require more computations. In both cases, a tricky part of the process is to ensure that the Σ matrix remains positive definite. A rewrite of Equation (8) with the Cholesky decomposition $\Sigma =L^{t}LK$ allows this condition to be satisfied by design and is the preferred choice. Another option to get an approximate value is to use Equation (7) after diagonalizing the covariance matrices.

In regard to the Riemannian metric $g_{w}$ which induces the Wasserstein distance [20], for Gaussian densities, the explicit expression of the distance is the following:

(10) $$d_{W}\left(\theta ,\theta^{\prime }\right)=\left|\mu -\mu^{\prime }\right|+tr\left(\Sigma \right)+tr\left(\Sigma^{\prime }\right)-2tr\left(\sqrt{\Sigma^{\frac{1}{2}}\Sigma^{\prime }\Sigma^{\frac{1}{2}}}\right)$$

where |·| is the euclidean norm and $\Sigma^{\frac{1}{2}}$ is defined for a symmetric positive definite matrix Σ so that $\Sigma^{\frac{1}{2}}\cdot \Sigma^{\frac{1}{2}}=\Sigma$. We remark that, if $\Sigma =\Sigma^{\prime }$, the Wasserstein distance reduces to the Euclidean distance.

Otto [20] proved that, with respect to the Riemannian metric which induces the Wasserstein distance, the manifold of Gaussian densities has non-negative sectional curvature. We deduce that the Wasserstein metric is different from the Fisher–Rao metric. Indeed, for example, in the univariate case, the statistical manifold of Gaussian densities with the Fisher–Rao metric can be regarded as the upper half plane with the hyperbolic metric, which has negative curvature as it is well known.

Once a distance is defined, it can be used for clustering on a manifold. It is proven that the distance induced from Fisher–Rao metric and Wasserstein distance are in the more general class of Bregman divergences defined by a convex function [21]. For this class, a theorem states [22] that the centroid for a set of n points $\theta_{i},i=1,2,\cdots ,n$ in the statistical manifold of the Gaussian densities is the Euclidean mean $\frac{1}{n}\sum_{i=1}^{n}\theta_{i}$. We use this result in the next section where a *K*-mean shapes clustering algorithm is defined using geodesic distances.

## 3. Clustering of Shapes

We consider only planar objects, as for example a flat fish or a section of the skull. The shape of the object consists of all information invariant under similarity transformations, that is translations, rotations and scaling [4]. Data from a shape are often realized as a set of points. Many methods allow to extract a finite number of points, which are representative of the shape and are called landmarks. One way to compare shapes of different objects is to first register them on some common coordinate system for removing the similarity transformations [2,23]. Alternatively, Procrustes methods [24] may be used in which objects are scaled, rotated and translated so that their landmarks lie as close as possible to each other with respect to the Euclidean distance.

Suppose we are given a planar shape configuration, *S*, consisting of a fixed number *K* of labeled landmarks

$$S=\left\{\mu_{1},\mu_{2},\cdots ,\mu_{K}\right\}$$

with generic element $\mu_{k}=\left\{\mu_{k1},\mu_{k2},\right\}$ for k=1,⋯,K. Following Gattone et al. [10], the *k*-th landmark, for k=1,⋯,K, may be represented by a bivariate Gaussian density as follows:

(11) $$f\left(x|\theta_{k}=\left(\mu_{k},\Sigma_{k}\right)\right)={\left(2\pi \right)}^{-1}{\left(\det\Sigma_{k}\right)}^{-\frac{1}{2}}\exp\left\{-\frac{1}{2}{\left(x-\mu_{k}\right)}^{T}\Sigma_{k}^{-1}\left(x-\mu_{k}\right)\right\}$$

with *x* being a generic 2-dimensional vector and $\Sigma_{k}$ given by

(12) $$\Sigma_{k}=\mathrm{diag}\left(\sigma_{k1}^{2},\sigma_{k2}^{2}\right)$$

where $\sigma_{k}^{2}=\left(\sigma_{k1}^{2},\sigma_{k2}^{2}\right)$ is the vector of the variances of $\mu_{k}$.

We remark that, in the previous representation, the means represent the geometric coordinates of the landmark and capture uncertainties that arise in the landmark placement. The variances are hidden coordinates of the landmark and reflect its natural variability across a population of shapes. Equation (11) allows assigning to the *k*th landmark the coordinates $\theta_{k}=\left(\mu_{k},\sigma_{k}\right)$ on the four-dimensional manifold which is the product of two upper half planes.

Let *S* and $S^{\prime }$ two planar shapes registered on a common coordinate system using Procrustes method. We parameterize them as follows: $S=(\theta_{1},\cdots ,\theta_{K})$ and $S^{\prime }=\left(\theta_{1}^{\prime },\cdots ,\theta_{K}^{\prime }\right)$.

The distances between landmarks allow defining a distance of the two shapes *S* and $S^{\prime }$. Precisely, a shape metric for measuring the difference between *S* and $S^{\prime }$ can be obtained by taking the sum of the geodesic distances between the corresponding landmarks, according to the following definition:

(13) $$D\left(S,S^{\prime }\right)=\sum_{k=1}^{K}d\left(\theta_{k},\theta_{k}^{\prime }\right)$$

Please note that this expression is not the geodesic distance on the product manifold that one would have expected from the landmark model. This last distance is given by:

(14) $$D\left(S,S^{\prime }\right)=\sqrt{\sum_{k=1}^{K}d{\left(\theta_{k},\theta_{k}^{\prime }\right)}^{2}}$$

and is a $L^{2}$ distance instead of Equation (13) that is $L^{1}$. It turns out that, according to simulations done, the $L^{1}$ approach is more robust and gives all the time better clusterings.

Then, a classification of shapes, using in turn, as distance *d*, the distance $d_{F}$ induced from Fisher–Rao metric and the Wasserstein distance $d_{W}$, can be done following the standard methodology. In particular, the *K*-means clustering procedure allows the variances to vary step by step in each cluster fitting better real shape data.

## 4. *K*-Means Clustering Algorithm

The proposed shape distances are implemented in two different *K*-means algorithms: Type I and Type II. While in the Type I algorithm the landmark coordinates variances are assumed isotropic across the clusters, in Type II the variances are allowed to vary among the clusters.

Our task is clustering a set of *n* shapes, $S_{1},S_{2},\cdots ,S_{n}$ into *G* different clusters, denoted as $C_{1},C_{2},\cdots ,C_{G}$.

### 4.1. Type I Algorithm

1. *Initial step:*
  Compute the variances of the *k*-th landmark coordinates $\sigma_{k}^{2}=\left(\sigma_{k1}^{2},\sigma_{k2}^{2}\right)$, for k=1,⋯,K.
  Randomly assign the *n* shapes, $S_{1},S_{2},\cdots ,S_{n}$ into *G* clusters, $C_{1},C_{2},\cdots ,C_{G}$.
  For g=1,⋯,G, calculate the cluster center $c_{g}=\left(\theta_{1}^{g},\cdots ,\theta_{K}^{g}\right)$ with *k*-th component $\theta_{k}^{g}=\left(\mu_{gk},\sigma_{k}^{2}\right)$ obtained as $\theta_{k}^{g}=\frac{1}{n_{g}}\sum_{i\in C_{g}}\theta_{k}^{i}$, where $n_{g}$ is the number of elements in the cluster $C_{g}$ and $\theta_{k}^{i}$ is the *k*-th coordinate of $S_{i}$ given by $\theta_{k}^{i}=\left(\mu_{ik},\sigma_{k}^{2}\right)$.
2. *Classification:*
  For each shape $S_{i}$, compute the distances to the *G* cluster centers $c_{1},c_{2},\cdots ,c_{G}$.
  The generic distance between the shape $S_{i}$ and the cluster center $c_{g}$ is given by:
  $$D\left(S_{i},c_{g}\right)=\sum_{k=1}^{K}d\left(\theta_{k}^{i},\theta_{k}^{g}\right)$$
  where the distance *d* could be the distance $d_{F}$ induced from Fisher–Rao metric or the Wasserstein distance $d_{W}$.
  Assign $S_{i}$ to cluster *h* that minimizes the distance:
  $$D\left(S_{i},c_{h}\right)=\min_{g}D\left(S_{i},c_{g}\right).$$
3. *Renewal step:*
  Compute the new cluster centers of the renewed clusters $c_{1},\cdots ,c_{G}$.
  The *k*-th component of the *g*-th cluster center $c_{g}$ is defined as $\theta_{k}^{g}=\frac{1}{n_{g}}\sum_{i\in C_{g}}\theta_{k}^{i}$.
4. Repeat Steps 2 and 3 until convergence [22].

### 4.2. Type II Algorithm

1. *Initial step:*
  Randomly assign the *n* shapes, $S_{1},S_{2},\cdots ,S_{n}$ into *G* clusters, $C_{1},C_{2},\cdots ,C_{G}$.
  In each cluster, compute the variances of the *k*-th landmark coordinates $\sigma_{gk}^{2}=\left(\sigma_{gk_{1}}^{2},\sigma_{gk_{2}}^{2}\right)$, for k=1,⋯,K and g=1,⋯,G.
  Calculate the cluster center $c_{g}=\left(\theta_{1}^{g},\cdots ,\theta_{K}^{g}\right)$ with *k*-th component $\theta_{k}^{g}=\left(\mu_{gk},\sigma_{gk}^{2}\right)$ obtained as $\theta_{k}^{g}=\frac{1}{n_{g}}\sum_{i\in C_{g}}\theta_{k}^{i}$ for g=1,⋯,G, where $n_{g}$ is the number of elements in the cluster $C_{g}$ and $\theta_{k}^{i}=\left(\mu_{ik},\sigma_{gk}^{2}\right)$ for $i\in C_{g}$.
2. *Classification:*
  For each shape $S_{i}$, compute the distances to the *G* cluster centers $c_{1},c_{2},\cdots ,c_{G}$.
  The generic distance between the shape $S_{i}$ and the cluster center $c_{g}$ is given by:
  $$D\left(S_{i},c_{g}\right)=\sum_{k=1}^{K}d\left(\theta_{k}^{i},\theta_{k}^{g}\right)$$
  where the distance *d* could be the distance $d_{F}$ induced from Fisher–Rao metric or the Wasserstein distance $d_{W}$.
  Assign $S_{i}$ to cluster *h* that minimizes the distance:
  $$D\left(S_{i},c_{h}\right)=\min_{g}D\left(S_{i},c_{g}\right).$$
3. *Renewal step:*
  Update the variances of the *k*-th landmark coordinates in each cluster by computing $\sigma_{gk}^{2}=\left(\sigma_{gk_{1}}^{2},\sigma_{gk_{2}}^{2}\right)$, for k=1,⋯,K and for g=1,⋯,G.
  Calculate the new cluster centers of the renewed clusters $c_{1},\cdots ,c_{G}$.
  The *k*-th component of the *g*-th cluster center $c_{g}$ is defined as $\theta_{k}^{g}=\frac{1}{n_{g}}\sum_{i\in C_{g}}\theta_{k}^{i}$.
4. Repeat Steps 2 and 3 until convergence [22].

## 5. Numerical Study

The purpose of the simulation study was to evaluate the cluster recovery of the proposed shape *K*-means algorithm and to test its sensitiveness with respect to different shape distances defined on the manifold of the probability distributions. The shapes were simulated according to a Gaussian perturbation model where the *i*th configuration is obtained as follows:

(15) $$X_{ig}=\left(\mu_{g}+E_{i}\right)\Gamma_{i}+1_{K}\gamma_{i}^{T}$$

where

- $E_{i}$ are zero mean K×2 random error matrices simulated from the multivariate Normal distribution with covariance structure $\Sigma_{E}$;
- $\mu_{g}$ is the mean shape for cluster *g*;
- $\Gamma_{i}$ is an orthogonal rotation matrix with an angle θ uniformly produced in the range [0,2π]; and
- $\gamma_{i}^{T}$ is a 1×2 uniform translation vector in the range [−2,2].

Three types of covariance structures are considered:

- Isotropic with $\Sigma_{E}=\sigma I_{K}⊗\sigma I_{2}$
- Heteroscedastic with $\Sigma_{E}=\mathrm{diag}\left[\sigma_{1},\sigma_{2}\cdots ,\sigma_{K}\right]⊗\sigma I_{2}$
- Anisotropic with $\Sigma_{E}=\sigma I_{K}⊗\mathrm{diag}\left[\sigma_{x},\sigma_{y}\right]$ with $\sigma_{x}\neq \sigma_{y}$

The data were generated from the model in Equation (15) with sample size n=100 and the number of clusters equal to G=2. The mean shapes in each cluster were taken from the rat calvarial dataset [1] corresponding to the skull midsagittal section of 21 rats collected at ages of 7 and 14 days. In the isotropic case, σ was equal to 13. In the heteroscedastic case, the values of $\sigma_{1},\sigma_{2},\cdots ,\sigma_{K}$ were equal to 13 for 3 randomly chosen landmarks and equal to 1.3 for the remaining 5 landmarks of each shape. Finally, the anisotropic case was simulated by setting $\sigma_{x}=13$ and $\sigma_{y}=1.3$ in one cluster and $\sigma_{x}=1.3$ and $\sigma_{y}=13$ in the other cluster.

**Figure 1:**
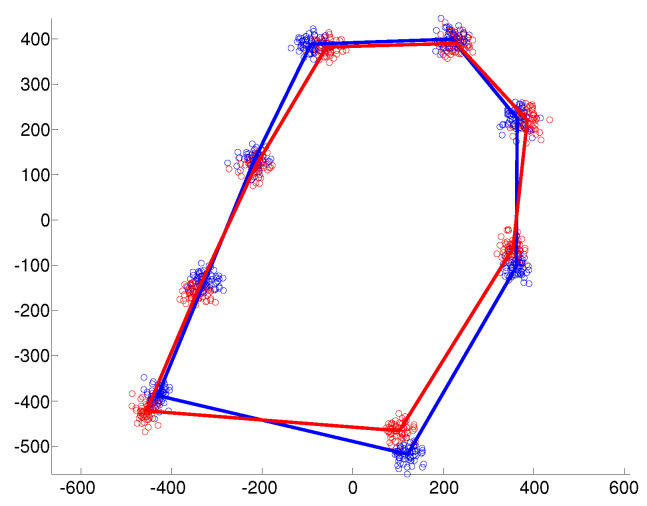
Independent spherical variation around each mean landmark.

**Figure 2:**
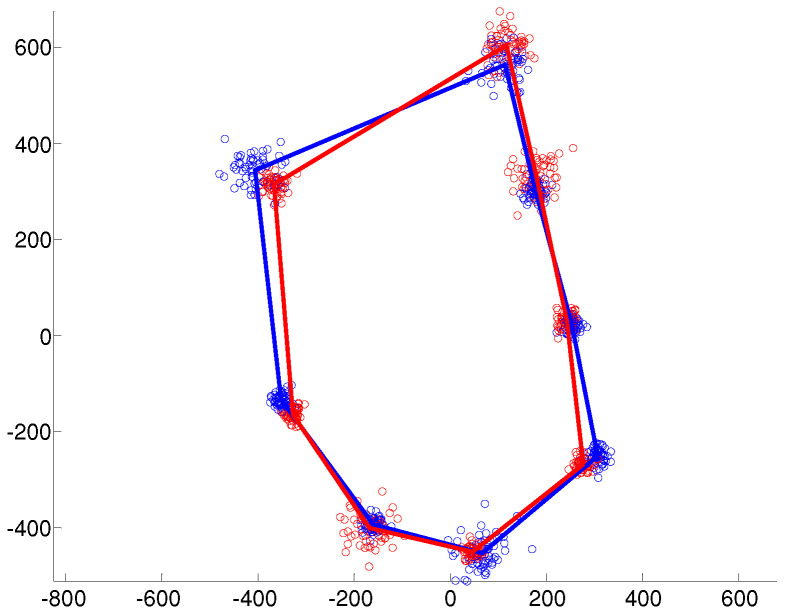
Heteroscedastic variation around each mean landmark.

**Figure 3:**
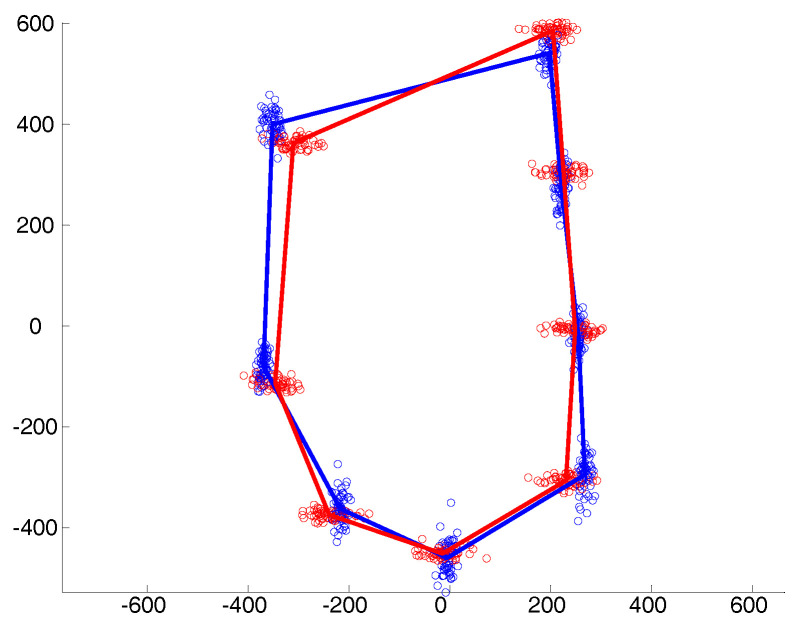
Anisotropy in the *x* and *y* directions around each mean landmark.

Examples of the simulated data under the different covariance structures are provided in Figure 1, Figure 2 and Figure 3. In the isotropic case (Figure 1), all landmark coordinates exhibit the same independent spherical variation around the mean. In the heteroscedastic case (Figure 2), the spherical variation is allowed to vary between the landmarks and the clusters. Finally, Figure 3 shows the anisotropic case where the variability of the landmark coordinates is different in the horizontal and vertical directions.

The shape *K*-means algorithm is implemented by using:

- The Fisher–Rao distance under the round Gaussian model representation (dr) where the variance is considered as a free parameter, which is isotropic across all the landmarks
- The Fisher–Rao distance under the diagonal Gaussian model representation (dd1 (Type I *K*-means algorithm) and dd2 (Type II *K*-means algorithm))
- Wasserstein distance (dp)

For each covariance structure, we simulated 150 samples and, for each sample, we computed the adjusted Rand index [25] of each clustering method. The adjusted Rand index is a measure of agreement between two partitions. It ranges from about 0 when the compared partitions are completely random to 1 when they are equal. The index is generally used as a measure of cluster recovery, the closer the index to 1 the better the clustering results.

**Figure 4:**
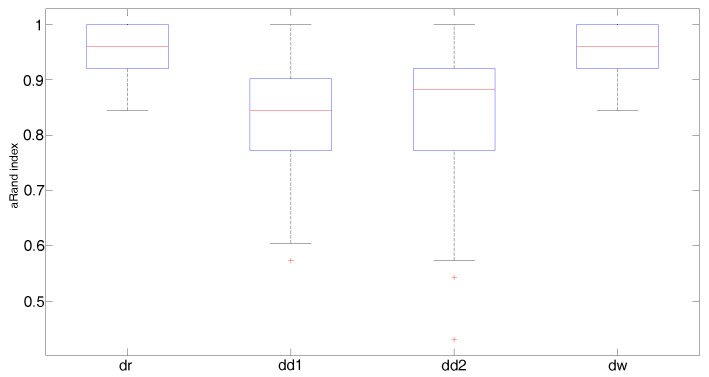
Isotropic case: Boxplots of the adjusted Rand index over 150 simulated samples for each clustering method; aRand index median values are 0.96 (dr), 0.85 (dd1), 0.88 (dd2), 0.96 (dw).

**Figure 5:**
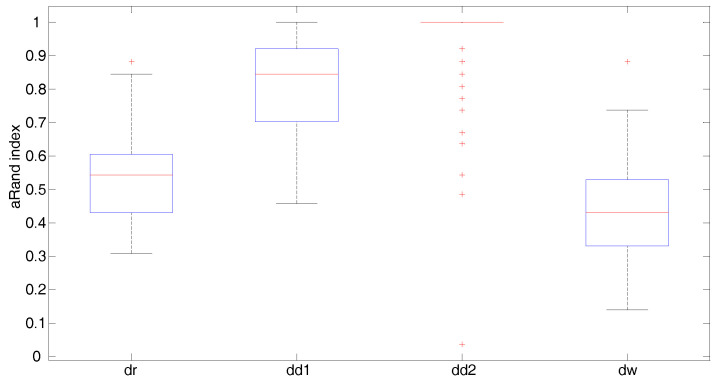
Heteroscedastic case: Boxplots of the adjusted Rand index over 150 simulated samples for each clustering method; aRand index median values are 0.54 (dr), 0.84 (dd1), 1.00 (dd2), and 0.43 (dw).

**Figure 6:**
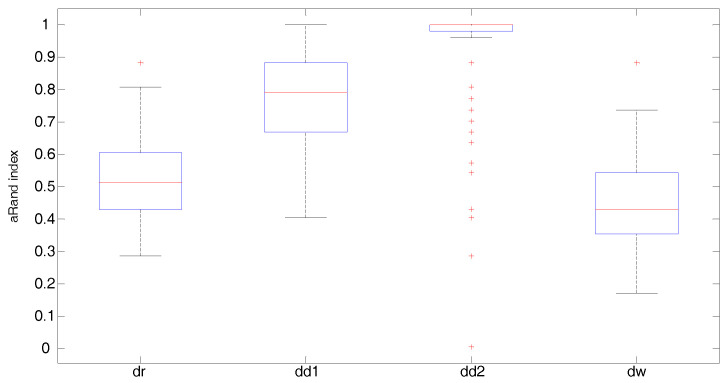
Anisotropic case: Boxplots of the adjusted Rand index over 150 simulated samples for each clustering method; aRand index mean values are 0.51 (dr), 0.79 (dd1), 1 (dd2), and 0.44 (dw).

Figure 4, Figure 5 and Figure 6 display the boxplots of the adjusted Rand index over 150 simulated samples for each clustering method. Outliers are plotted individually with the + symbol. When the covariance structure is isotropic (Figure 4), all distances show a similar behavior. In particular, the Fisher–Rao distance with round Gaussian distribution (dr) and the Wasserstein distance (dw) yield the best clustering results with median values of the adjusted Rand index both equal to 0.96 versus 0.85 and 0.88 obtained by the diagonal Gaussian distribution with Type I (dd1) and Type II (dd2) algorithms, respectively. In the heteroscedastic setting (Figure 5), both the Fisher–Rao with the round Gaussian distribution (median adjusted Rand index equal to 0.54) and the Wasserstein distance (median adjusted Rand index equal to 0.43) perform poorly in comparison to the Fisher–Rao distance based on the diagonal distribution. As expected, the models which take into account different landmark variances (dd1-Type I algorithm) and also differences in the variances between the clusters (dd2-Type II algorithm) show a very good behavior with median values of the adjusted Rand index equal to 0.84 and 1, respectively. A very similar pattern is observed when anisotropy is also added in the covariance structure (Figure 6). As expected, the Type II algorithm significantly increases the computational time needed to meet the convergence condition since in each iteration both the means and the variances of the cluster centers have to be updated. On average, the Type II algorithm’s running time is approximately 20 times longer than Type I.

## 6. Conclusions

In this study, Information Geometry was used as a useful tool in the area of shape clustering. We first described a shape representing each landmark by a Gaussian model using the mean and the variance as coordinates, reflecting the geometrical shape of the configuration and the variability across a family of patterns, respectively. Within this framework, we considered the Fisher–Rao and the Wasserstein metric for quantifying the difference between two shapes.

Two version of the Fisher–Rao metric were proposed, depending on how the variances in the data are employed. In one case (round Gaussian distribution model), the variance was considered a free parameter that is isotropic across all the landmarks. In the second case, the isotropic assumption was relaxed allowing the variances to vary among the landmarks (diagonal Gaussian distribution model).

The results of the numerical study have shown that the violation of the isotropic assumption on the landmarks variability may cause a severe loss in the clustering recovery. Indeed, this assumption is rarely satisfied in practice where it is regularly seen that landmarks have different variances. In such a case, the relative importance among landmarks must be taken into account in the similarity measure adopted in the clustering algorithm. The proposed geodesic distance under the diagonal Gaussian model representation is able to face this problem. A further assumption that may be violated is that in all clusters the landmarks coordinates have a common covariance matrix. To cope with this issue, a new *K*-means shape algorithm was implemented that allows for differences among the clusters in the landmark coordinates variability.

Other extensions of the current work deserve further investigation, for example, the use of geodesics in the case of the general multivariate Gaussian model and considering more general shape measures, such as α-divergences.
